# A Successful Case of Ovariotomy

**Published:** 1857-03

**Authors:** Samuel Long

**Affiliations:** Springfield, Ill.


THE NORTH-WESTERN

MEDICAL AND SURGICAL JOURNAL.

(NEW SERIES.)

VOL. VI. MARCH, 1857. NO. 3.

### ORIGINAL COMMUNICATIONS.

ARTICLE I.—*A Successful Case of Ovariotomy.* By SAMUEL LONG, M.D. Springfield, Ill.

On the 15th day of June, 1856, I was summoned to visit Mrs. D. aged twenty-six, married; a small woman; weight, about one hundred and fifteen pounds. She had been under the care of several physicians of this place, and had taken a great deal of medicine for her malady, which at first was supposed to be dropsy of the abdomen, but of late had been pronounced ovarian disease.

According to her statement, she first became aware of its existence immediately after the birth of her child, eighteen months before I was called to visit her. The tumor was then of considerable size, occupying the left hypochondrium; soft, free from pain, and gave her no uneasiness whatever. During the first twelve months after the discovery of its existence, her health was very good, but latterly she was oppressed by the size of the tumor. She was becoming thin; her breathing was hurried and difficult, and the tumor steadily increased in size.

I gave my opinion to herself and husband, that her disease, as had been stated by her physician was ovarian; and that it was useless to attempt to relieve by medicine. I then stated to them plainly the nature of the disease, and, as I conceived, the only remedy—ovariotomy. As the case was one not requiring immediate operation, and hoping that nature might perhaps relieve herself, it was determined to postpone the operation for the present.

During the next two months (July and August) the tumor grew very little, but from the middle of August to the middle of September, its growth was very rapid, so that Mrs. D. could not lie down with any degree of comfort. The whole abdomen was filled, and almost as tight as a drum; nor did change of posture make any difference in the size of the tumor. It could now be felt very plainly through the abdominal walls, and was evidently multilocular, with some portions much harder than others. The lady was very anxious to have the operation performed; and, as it was evident that she could not long survive if 'let alone,' as she was emaciating very fast, losing her appetite and her strength, it was determined to remove the tumor by an incision in the walls of the abdomen.

As such opinion was confirmed on consultation with Dr. M. Helm, Dr. W. S. Wallace, and Drs. Lord & Fowler, all eminent physicians of Springfield, on the 20th day of September, 1856, assisted by the above-named gentlemen, I performed the operation, the bladder being previously emptied.

Operation commenced at 10½ o'clock. Patient was brought under the influence of chloroform; the administration of which was interrupted several times by the vomiting of the patient. First, made an incision in the *linea alba*, about three inches in length, below the umbilicus, which exposed the tumor; I then introduced my hand and discovered, after a careful exploration, that there were strong attachments to the peritoneum, to the left of the umbilicus, above, for a space two or three inches square; this was the only place of attachment so far as we then could discover. The incision was then continued to the sternum, leaving the umbilicus to the right; the length of the wound being sixteen inches. The attachments were broken up partly with the hand and partly with the knife. The tumor was immediately lifted out of the abdomen, and was found attached on the left side by its pedicle to the broad ligament of the uterus. The pedicle, which was half an inch in diameter, was transfixed by a needle armed with a double-headed ligature, and then cut off, half an inch above the ligature.

The tumor consisted of several large cyst, filled with a thick, honey-like fluid, and of several smaller ones of the consistency of butter—all united together. Its weight was twenty pounds eight ounces. On taking it out of its place, the uterus, the intestines (pressed towards the spine), the stomach, the liver, and spleen, were all brought into view; all seemed healthy and of the natural size. The play of the diaphragm, at each inspiration and expiration could be seen. The ligature of the pedicle was brought out at the lower part of the wound. The incision in the abdomen was then brought together by the common interrupted suture, three-fourths of an inch apart; the intervening spaces being approximated by straps of adhesive plaster. The whole was then covered with a light compress, and a bandage placed around the body. The intestines gave no trouble, as they remained close to the spine. During the whole operation not half an ounce of blood was lost.

One hour after operation—pulse quick and small; skin cool; no reaction. Four hours after operation — pulse more full, about eighty, compressible; reaction has begun. The patient says she feels very well; cannot feel the wound; can scarcely realize that she has had her burden removed. Ten hours after operation—slight thirst; pulse eighty-five, full and compressible; feels 'very comfortable.' Sulph. morph. grain half.

*Second Day, Eight o'Clock A.M.*—Rested well last night; pulse and heat of skin natural; occasionally sick at stomach; prescribed small pieces of ice *ad libitum;* feels 'well.'

*Five o'Clock P.M.*—Vomited at 1 o'clock; pulse one hundred and fifteen, fine; 'feels comfortable.' Menstruation appeared this afternoon.

*Third Day, Eight o'Clock A.M.*—Pulse one hundred and thirty, quick and fine; did not rest well last night; vomited several times during night; feels slightly sore. At night, same as morning; vomited several times during day; menstruation continues.

*Fourth Day*—Rested badly; pulse same as yesterday; vomited occasionally; wound looks well; menstruation continues.

*Fifth Day*—Slept well last night; pulse one hundred and twenty; feels no soreness.

*Eight o'Clock P.M.*—Feels very comfortable; has vomited none since last night; diet, weak wine whey and custard; menstruation ceased to-day.

*Sixth Day*—Same as yesterday. Continued diet.

*Seventh Day*—Rather restless this morning; pulse one hundred and twenty; skin cool. Ordered glyster of warm water, soap, and molasses, which caused the bowels to move well for the first time since operation. Feels much better in the evening.

*Eighth Day*—Passed a very comfortable night; pulse about same; appetite ravenous. Wound looks well; seems healed in its superior half by first intention; the inferior half is healing by suppuration. Patient says she feels as well as she ever did.

*Ninth Day*—Passed a very comfortable night; pulse about same. Ordered injection as before. Bowels moved well. Feels considerable pain in bladder at night.

*Tenth Day*—Pulse one hundred and twenty; appetite good; suffers still pain in bladder. The wound which apparently had healed by first intention, is now suppurating throughout its whole extent.

*Eleventh Day*—Rested well last night; same as yesterday. Ordered injection, which did not operate well.

*Twelfth Day*—Same as yesterday.

*Thirteenth Day*—Rested well last night; at noon, suffered a good deal of pain and uneasiness in bowels; very restless.

*Eight o'Clock P.M.*—Gave table-spoonful and a half of castor oil.

*Fourteenth Day*, *Three o'Clock A.M.*—Bowels moved well; relieved of pain and restlessness; had three or four motions of bowels to-day; weak and nervous. About 10 o'clock P.M. bowels moved freely, causing considerable exhaustion—patient thought she was dying. Gave ℥ss vin. Oport, gtt. xv. elixir of opium. Soon revived and slept. Ligature of the pedicle came away about midnight, followed by two or three ounces of pure pus.

*Fifteenth Day*—Feels very comfortable; slept soundly since midnight; appetite very good; light diet; wound is healing well.

From this date, Mrs. D. continued to improve rapidly. Wound suppurating freely until the fourth or fifth week, when it was almost entirely healed. The ligatures in the wound were all taken away by the tenth day. At the present time, January 17th, 1857, Mrs. D. says she is as well as she ever was, and as strong, and I should judge one of the happiest, as well as one of the most grateful of women. Menstruation returned the first week in this month (Jan. 1857), naturally.

For three weeks, Mrs. D. according to direction, laid upon her back, turning neither to the right nor left side. From the moment of the operation to the present time, there has been scarcely any soreness of the abdomen, and no tenderness on pressure. The urine was evacuated for three weeks with the catheter, every six or eight hours. At the commencement of the second week, the urine was tinged with blood, and the evacuation of it caused considerable pain; this lasted for one week, when it returned to its normal condition. A few hours after the operation, half a grain of morphia was given, but it (and all other preparations of opium) caused so much nausea, that it was soon discontinued, and no other remedies were given, save one or two doses of castor oil, and injections; repeated two or three times. Her only cause of complaint was (as she playfully said,) that I kept her upon her back so long.

The favorable features in this case, before the operation, were, that the patient's constitution was good; there never had been any pain caused by the tumor, nor any soreness of the abdomen, and no peritonitis; therefore, I thought there were probably no adhesions. It was not until after the tumor was removed that she told me she had had, about seven months before the operation, considerable pain in her side, just where the only adhesions were found.

Of the propriety of the operation of Ovariotomy, enough has been written of late years. I am only too happy to record one more successful one of this truly hazardous and dangerous operation.

